# The Timing of the Melatonin Onset and Phase Angle to Sleep Onset in Older Adults after Uncontrolled vs. Controlled Lighting Conditions

**DOI:** 10.3390/clockssleep5030026

**Published:** 2023-06-25

**Authors:** Arturo Arrona-Palacios, Jung-Hie Lee, Charles A. Czeisler, Jeanne F. Duffy

**Affiliations:** 1 Division of Sleep and Circadian Disorders, Department of Medicine, Brigham and Women’s Hospital, Boston, MA 02115, USA; aarronapalacios@bwh.harvard.edu (A.A.-P.); jhielee@kangwon.ac.kr (J.-H.L.); charles_czeisler@hsm.harvard.edug (C.A.C.); 2 Division of Sleep Medicine, Harvard Medical School, Boston, MA 02115, USA; 3 Department of Psychiatry, Kangwon National University School of Medicine, Chunchon 200-947, Republic of Korea

**Keywords:** circadian phase, melatonin, adults, aging

## Abstract

The main aim of this study was to explore how melatonin onset timing and phase angle to bedtime in healthy older adults are impacted by prior light exposure. A total of 13 healthy older (ages 56–74) individuals were studied on two successive evenings. Prior to the first evening, the participants were in self-selected lighting conditions for the first 4–6 h of the day and then were in dim light (3 lux) until their scheduled bedtime. On the second day, individuals from Project A remained in the dim lighting conditions throughout the entire day but those in Project B were in more typical indoor lighting (~90 lux) throughout the day. On both evenings, hourly blood samples were collected and assayed for melatonin, and melatonin onset timing and phase angle to sleep onset was determined. Overall, melatonin onset was earlier and the phase angle was larger on Night 1 than on Night 2. In Project A there was no significant difference between melatonin onset on night 1 vs. night 2. However, in Project B melatonin onset was significantly later on Night 2 (in typical indoor lighting) than on Night 1 (in dim lighting). Our results suggest that in older people, uncontrolled bright light early in the day did not impact the timing of dim light melatonin onset (DLMO) when assessed later that same evening. However, in older adults, exposure to ordinary room light during melatonin phase assessment appeared to suppress melatonin, leading to a later observed time of melatonin onset, as has been reported previously for young adults.

## 1. Introduction

The pineal gland activity is low during the day and then during the evening, it synthesizes the hormone melatonin that is then released into the blood circulation during the night. Melatonin thus serves as an endocrine signal of darkness. The timing of melatonin production is regulated through a pathway from the suprachiasmatic nuclei (SCN), the central circadian pacemaker. The SCN receives information about the environmental light–dark cycle through a pathway from the retina, and in turn, passes this information to the pineal gland [1]. Exposure to light during the biological night when melatonin is being secreted can acutely suppress melatonin [2,3].

Because circulating melatonin concentrations are low during the day and then abruptly rise in the evening when secretion begins [4]; the time at which this evening rise occurs has become a convenient measure of the central circadian phase in research studies [5,6,7,8,9]. More recently, the dim light melatonin onset (DLMO) has also been assessed in patients with circadian rhythm sleep-wake disorders in order to determine whether those patients have abnormal timing of their circadian rhythms compared to control participants [7,10,11,12]. The stability of DLMO from day to day and how measurement conditions may impact DLMO timing are important to understand when using this research tool in clinical applications [13]. Because light can have an important effect on melatonin production when assessing DLMO in patients either in the home environment [14] or in controlled laboratory conditions [15], understanding how lighting during and immediately prior to the DLMO sample collection impacts DLMO timing is necessary. In young adults, even indoor levels of light during DLMO sampling have been shown to suppress melatonin secretion and delay melatonin onset compared to collection under dim lighting [16,17]. In addition to this acute effect of light on circulating melatonin concentrations, there is also evidence from young adults of adaptation or sensitization to light, where brighter light exposure on the day or days prior to melatonin sampling changes the magnitude of responses to light exposure during sampling [18,19].

We and others have reported that the timing of the melatonin phase in healthy older adults when assessed in controlled laboratory lighting conditions is earlier than in young adults and that the relative timing between melatonin and sleep timing is significantly different between older and young adults [20,21,22]. Moreover, most previous studies of melatonin responses to light have been conducted in young adults. Therefore, the main objective of this analysis was twofold: first, to explore how DLMO is impacted by exposure to uncontrolled vs. controlled lighting early on the day of assessment; and second, to explore how melatonin onset is impacted by different indoor light levels (dim vs. room lighting) in healthy older adults.

## 2. Results

**Figure 1:**
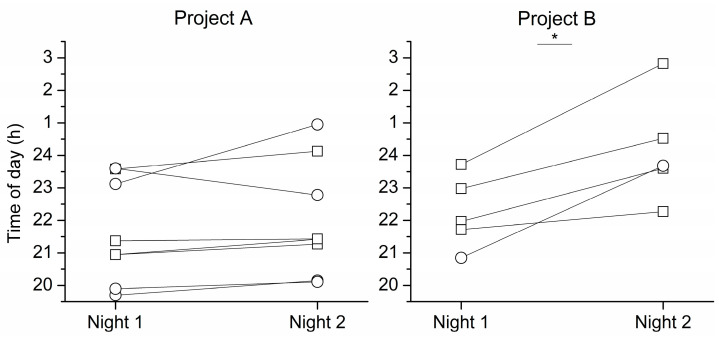
(**Left**): Timing of dim light melatonin onset (DLMO) on Night 1 (3 lux) and Night 2 (3 lux) for Project A (*p* = 0.18); and (**Right**): melatonin onset on Night 1 (3 lux) and Night 2 (90 lux) for Project B (*p* = 0.01). Each symbol represents one participant, with their DLMO on Night 1 connected to their DLMO on Night 2. Open squares represent men (*n* = 8) and open circles represent women (*n* = 5). The differences between Night 1 and Night 2 in Project A and Project B were analyzed separately with paired *t*-test. * *p* ≤ 0.05.

The total number of participants included in the final analyses was 13, with 8 participants in Project A and 5 participants in Project B (see Figure 1). This included 5 women and 8 men ranging in age from 56 to 74 years (64.30 ± 5.25 years), and their average bedtime in the week prior to study while maintaining a self-selected 8 h sleep schedule was 22:57 ± 0:32 h.

For Project A, the phase angle between DLMO and bedtime was not significantly different between Night 1 and Night 2 (Night 1: −2:24 ± 1:31 h vs. Night 2: −2:01 ± 1:48 h, *t* = −1.48, *p* = 0.18), whereas for Project B, phase angle between melatonin onset and bedtime was significantly shorter on Night 2 than Night 1 (Night 1: −1:31 ± 1:41 h vs. Night 2: 0:24 ± 2:21 h, *t* = −4.15, *p* = 0.01). Furthermore, for Project A, no significant difference was found for DLMO timing between Night 1 and Night 2 (Night 1: 20:38 ± 1:35 h vs. Night 2: 21:02 ± 1:46 h, *t* = −1.48, *p* = 0.18), although the average DLMO on Night 2 was 20 min later than on Night 1. For Project B, the melatonin onset on Night 2 was significantly later than Night 1 by nearly 2 h (Night 1: 21:14 ± 1:06 h vs. Night 2: 23:10 ± 1:41 h, *t* = −4.15, *p* = 0.01, see Figure 1).

Multivariate analysis of variance (MANOVA) confirmed these findings. The phase angle of DLMO to bedtime was not significantly different on Night 1 between Project A and Project B (Night 1: Project A: −2:24 ± 1:31 h vs. Project B: −1:31 ± 1:41 h, mean difference = −0:52 h, *p* = 0.35), whereas on Night 2 Project A had a significantly longer phase angle between melatonin onset and bedtime than Project B (Night 2: Project A: −2:01 ± 1:48 h vs. Project B: 0:24 ± 2:21 h, mean difference = −2:25 h, *p* = 0.05). In addition, there was no significant difference in the timing of DLMO on Night 1 between Project A and Project B (Night 1: Project A: 20:38 ± 1:35 h vs. Project B: 21:14 ± 1:06 h, mean difference 0:36 h, *p* = 0.47). However, on Night 2, melatonin onset for Project A was two hours earlier than Project B (Night 2: Project A: 21:02 ± 1:46 h vs. Project B: 23:10 ± 1:41 h, mean difference 2:08 h, *p* = 0.05).

## 3. Discussion

The main objective of this study was to explore how DLMO and phase angle between DLMO and bedtime are impacted by prior uncontrolled lighting exposure on the day of assessment compared to when lighting is controlled throughout the day of assessment, as well as to compare how melatonin onset is impacted when assessed under different light levels (dim vs. room lighting) in healthy older adults. Our results indicated that for Project B, exposure to different lighting during melatonin assessment impacted melatonin onset, with onset significantly later when assessed in room light levels than in dim light, presumably due to melatonin suppression as represented in Figure 1. These results obtained from healthy older adults are consistent with findings from young adults [16,17,18,19,23]. Moreover, the phase angle in healthy older adults in this study was comparable to young adults [23]. Interestingly, our finding of the range of DLMO timing in adults aged 56–74 coincides with a recent review analyzing DLMO across the ages [24].

Studies in young adults have shown that prior exposure to light during the day reduces the suppressive effect of light on melatonin at night Nevertheless, in our study with healthy older adults the finding that DLMO timing in Project A did not change significantly between Night 1 and Night 2 as shown in Figure 1, despite the differences in lighting during the first half of the day, is also of interest. On Day 1 these participants woke at home and spent the first 4–6 h of their day in uncontrolled lighting at home and on their travel to the laboratory for their study. They then entered controlled dim lighting for the final ~10 h of their waking day. In contrast, on Day 2 they woke in the laboratory and spent the entire 16 h wake episode in dim lighting. Understanding how exposure to brighter light early in the day might impact the timing of DLMO assessed in the evening is of particular interest for researchers and clinicians who want to assess DLMO in patients where it may be impractical to have them in controlled lighting for an entire day.

In Project A, the average DLMO on Night 2 was later than on Night 1, as would be expected given that the intrinsic period of the central circadian pacemaker is longer than 24 h and that the participants were exposed to brighter light on the morning of Day 1, although the difference (20 min) did not reach statistical significance in this small study sample. Additional studies to determine how the duration, timing, and intensity of light on the day of DLMO assessment impacts DLMO and the duration of dim light necessary to assess DLMO with accuracy should be carried out.

In summary, our results suggest that the background lighting during sample collection for DLMO assessment is important for older, as well as young adults and that lighting of even modest indoor intensity can suppress and/or delay melatonin onset in older adults. Our findings also show that brighter uncontrolled lighting on the morning of DLMO assessment minimally interferes with DLMO timing as long as DLMO is assessed in dim light.

### 3.1. Limitations

Although this study expanded our understanding of the impact of prior uncontrolled light exposure on healthy older adults, there are several limitations. First, the analyses were carried out on existing data, and the studies were not originally designed to examine differences in DLMO depending on prior light exposure. In addition, the sizes of the two groups we compared were small and that likely influenced our finding in Project A that despite a difference in DLMO of 20 min between Night 1 and Night 2, it was not statistically significant. Furthermore, Project B did not have an equal sex distribution, and this could have had an impact on our findings. Finally, due to the small sample size, we were not able to examine potential seasonal differences that might influence light exposure, such as photoperiod or weather.

### 3.2. Future Directions

Future prospective studies should be powered to examine potential sex differences and differences related to the season. Studies from younger adults have found differences in DLMO timing related to the use of light-emitting devices, and no such data are yet available from older adults. A recent report found a genetic marker associated with light sensitivity [25], implying that there may be genetic factors that contribute to differences in circadian rhythms. Highlighting that future studies should explore how individual differences in light sensitivity may impact the accuracy of DLMO assessments and/or the light levels required during and prior to the assessment of DLMO.

## 4. Materials and Methods

### 4.1. Participants

Participants included in this analysis took part in one of two different projects: Project A (*n* = 8), a 32 day study investigating the impact of pre-sleep melatonin administration (on days following the baseline days reported here) [26,27] or Project B (*n* = 5), a 9 day study investigating the impact of light exposure on circadian timing [28,29]. The recruitment, screening, and admission day conditions of both studies were the same. A total of 34 participants took part in the studies. Fourteen participants were excluded from the present analysis because their melatonin samples were assayed at a different laboratory using a different assay methodology. Seven additional participants were excluded from the analysis because they did not have a complete data set (missing samples on Night 1 or Night 2). A total of 13 participants were included in the final analyses. Participants were recruited from the community using flyers and newspaper advertisements directed to people aged 55 and older. The medical screening included routine clinical tests on blood and urine, an electrocardiogram, a physical examination, and a medical history to rule out acute or chronic illnesses and medication use. All participants were in good psychological health as determined by the Geriatric Depression Scale, Mattis Dementia Rating Scale, Folstein Mini-Mental State Exam, the Minnesota Multiphasic Personality Inventory-2, and by clinical interview [30], and all were free from clinically significant sleep disorders as determined by all-night clinical polysomnography. None of the participants were under the care of a physician for any chronic medical condition, and none were regularly taking medications. They were instructed to abstain from caffeine, nicotine, alcohol, and all prescription and over-the-counter medications for three weeks before the study; compliance with this aspect of the study was verified upon admission to the laboratory by a comprehensive toxicological analysis of their urine.

### 4.2. Study Protocol

**Figure 2:**
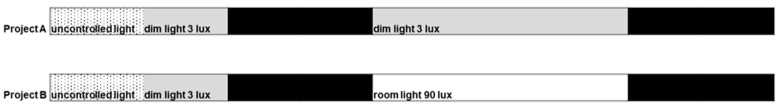
Protocol study of Project A and Project B baseline days. Dotted white bars represent uncontrolled lighting, grey bars represent dim light at 3 lux, white bar represent room light at 90 lux and black bars represent the 8-h scheduled in bed.

Both projects followed the same 2 day baseline procedures (with the exception of lighting conditions). Participants were admitted to the laboratory in the early afternoon of Day 1. They were scheduled to be in bed in the dark for 8 h on each of the 2 baseline nights (Figure 2), scheduled according to each participant’s average sleep times from the week immediately prior to the study (calculated from sleep diaries and confirmed with actigraphy data). Participants in both studies had structured baseline days consisting of regularly scheduled meals, a shower between breakfast and lunch, and neurobehavioral performance tests at approximately 2-h intervals throughout the day. After the baseline days, each of the studies followed different schedules which have been reported elsewhere [26,27,28,29].

### 4.3. Light Conditions

All lighting in the laboratory was provided by ceiling-mounted fluorescent lamps (T8 or T12 lamps with CCT of 4100K, Philips Lighting Eindhoven, The Netherlands) transmitted through ultraviolet (UV)-shielding ceiling filters (Lexan, GE Plastics, Pittsfield, MA, USA). All lighting was controlled by the experimenters at all times and participants had no access to any other lighting. On the day of admission to the study, ambient lighting was approximately 0.0087 W/m^2^ (3.3 lux) at 137 cm from the floor facing the walls and had a maximum of 0.048 W/m^2^ (15 lx) at 187 cm from the floor facing the ceiling anywhere in the room. Throughout the 8-h scheduled time in bed on Nights 1 and 2, all lighting was turned off. On the second baseline day, the ambient lighting in Project A was the same as on the admission day for the entire 16-h wake episode. In Project B, the lighting was approximately 0.23 W/m^2^ (~90 lx) at 137 cm from the floor facing the walls and had a maximum of 0.48 W/m^2^ (150 lx) at 187 cm from the floor facing the ceiling anywhere in the room (Figure 2).

### 4.4. DLMO and Melatonin Onset Assessment and Phase Angle to Sleep

Shortly after admission, an intravenous catheter was placed in the forearm vein of the participant by a research nurse. This was connected to a 12-foot tubing so that blood could be sampled from outside the room while the participant was sleeping. Blood was collected every 30–60 min and placed into tubes containing EDTA and then placed on ice for up to an hour before being centrifuged; the resulting plasma was frozen at −20 °C until assay. Plasma samples were assayed for melatonin using a radioimmunoassay (assay sensitivity 0.7 pg/mL; intra-assay coefficient of variation 12.1% at 16.5 pg/mL, 5.7% at 68.7 pg/mL; inter-assay coefficient of variation 13.2% at 17.3 pg/mL, 8.4% at 68 pg/mL; Pharmasan Labs, Osceola, WI, USA). For Project A, dim light melatonin onset (DLMO) was determined by linear interpolation between adjacent samples using a relative threshold. The relative DLMO threshold was the value at which the melatonin level exceeds two standard deviations (SD) of the average of three baseline values [5]. The relative DLMO threshold was calculated for the admission day (Night 1) for each participant and the same threshold was applied for the second day (Night 2) as well. In Project B, DLMO was assessed on Night 1 in the same way. On Night 2, we used the same threshold to calculate melatonin onset (although refrain from calling this and melatonin onset on “DLMO” because it was not assessed under dim light conditions). Phase angle was defined as the interval between DLMO or melatonin onset and bedtime (the average from the week prior to the study).

### 4.5. Statistical Analysis

Descriptive analysis was conducted to generate means and standard deviations (mean ± SD) for age, habitual bedtime, DLMO or melatonin onset, and phase angle of DLMO or melatonin onset to bedtime. For each participant, a comparison of melatonin onset timing and phase angle between Nights 1 and 2 was tested by a paired *t*-test. To compare between groups, DLMO or melatonin onset timing and phase angle were assessed using a multivariate analysis of variance (MANOVA) with Bonferroni correction. Data are presented as mean ± standard deviation unless otherwise mentioned.

## Data Availability

The data presented in this paper is available from the corresponding author in compliance with Mass General Brigham data sharing policies.
